# Predictors of 2-Year Incidence of Patient-Reported Urinary Incontinence After Post-prostatectomy Radiotherapy: Evidence of Dose and Fractionation Effects

**DOI:** 10.3389/fonc.2020.01207

**Published:** 2020-07-23

**Authors:** Andrea Bresolin, Elisabetta Garibaldi, Adriana Faiella, Domenico Cante, Vittorio Vavassori, Justina Magdalena Waskiewicz, Giuseppe Girelli, Barbara Avuzzi, Elisa Villa, Alessandro Magli, Barbara Noris Chiorda, Fernando Munoz, Giuseppe Sanguineti, Pietro Gabriele, Marco Gatti, Tiziana Rancati, Riccardo Valdagni, Nadia Di Muzio, Claudio Fiorino, Cesare Cozzarini

**Affiliations:** ^1^IRCCS Istituto Scientifico Ospedale San Raffaele, Medical Physics, Milan, Italy; ^2^Fondazione Centro San Raffaele, Milan, Italy; ^3^Istituto di Candiolo—Fondazione del Piemonte per l'Oncologia IRCCS, Radiotherapy, Turin, Italy; ^4^IRCCS Istituto Nazionale dei Tumori “Regina Elena,” Radiotherapy, Rome, Italy; ^5^Ospedale di Ivrea, Radiotherapy, Ivrea, Italy; ^6^Cliniche Gavazzeni-Humanitas, Radiotherapy, Bergamo, Italy; ^7^Comprensorio Sanitario di Bolzano, Radiotherapy, Bolzano, Italy; ^8^Ospedale degli Infermi, Radiotherapy, Biella, Italy; ^9^Fondazione IRCCS Istituto Nazionale dei Tumori, Radiotherapy, Milan, Italy; ^10^Azienda Ospedaliero Universitaria S. Maria della Misericordia, Radiotherapy, Udine, Italy; ^11^Ospedale Regionale Parini-AUSL Valle d'Aosta, Radiotherapy, Aosta, Italy; ^12^Fondazione IRCCS Istituto Nazionale dei Tumori, Prostate Cancer Program, Milan, Italy; ^13^Department of Oncology and Hemato-Oncology, University of Milan, Milan, Italy; ^14^Istituto Scientifico Ospedale San Raffaele, Radiotherapy, Milan, Italy; ^15^University Vita-Salute San Raffaele, Milan, Italy

**Keywords:** urinary incontinence, predictive models, prostatectomy, radiotherapy, prostate cancer

## Abstract

**Objective:** To investigate predictors of patient-reported urinary incontinence (PRUI) in the first 2 years after post-prostatectomy radiotherapy (PORT) with particular emphasis on possible dose-effect relationships.

**Patients and Methods:** Two-hundred-thirteen patients, whose clinical and dosimetric data were prospectively collected within a registered multi-institutional cohort study, underwent PORT with adjuvant (*n* = 106) or salvage (*n* = 107) intent with conventional (*n* = 123, prescribed dose to the prostatic bed: 66.6–79.8Gy in 1.8–2.0Gy/fr) or moderately hypo- (*n* = 90, 65.8–76.8Gy in 2.1–2.7Gy/fr) fractionation during the period 2011–2017. PRUI was evaluated through the ICIQ-SF questionnaire filled in at baseline and every 6 months thereafter. The analysis focused on three ICIQ-based clinically relevant endpoints: (a) very frequent leakage (FREQUENCY, ICIQ3 score >3), (b) moderate to severe amount of urine loss (AMOUNT, ICIQ4>2) (c) objective severe symptoms (OBJECTIVE, ICIQ3+4>5). Predictors of the incidence within 2 years for the three endpoints were investigated focusing only on patients without endpoint symptoms at baseline. A uni-variable logistic regression analysis was performed in order to determine the best dose metrics describing PRUI risk in terms of 2-Gy equivalent dose (EQD2) calculated with different α/β values reported in the literature (0.8, 3, 5Gy), and to identify the most significant clinical variables. Variables showing *p* < 0.20 at uni-variable analysis were entered into a backward stepwise multi-variable logistic regression analysis. Lastly, the goodness of fit and model calibration were evaluated and internally validated.

**Results:** Patients without symptoms at baseline experienced (a), (b), and/or (c) within 2 years in 41/130 (32%), 40/192 (21%), and 41/129 (32%) of the cases, respectively. EQD2 for α/β = 0.8Gy was the best dose metric associated with PRUI. Multi-variable analysis identified baseline incontinence levels as the strongest predictor for all endpoints (*p* < 0.006). Both FREQUENCY and OBJECTIVE were significantly influenced also by EQD2(α/β = 0.8Gy). The goodness of fit was excellent, as was the calibration; internal calibration confirmed apparent performance.

**Conclusion:** Baseline mild urinary incontinence symptoms strongly modulate the 2-year risk of PRUI. In addition, FREQUENCY is characterized by a marked dose-effect relationship also influencing the trend of OBJECTIVE, with results more reliable than AMOUNT as an objective index. A strong impact of fractionation on severe PRUI after post-prostatectomy radiotherapy also emerged.

## Introduction

Urinary toxicity is a common side effect of radiotherapy for prostate cancer (PCa), despite the modern intensity-modulated (IMRT) delivery techniques and image-guidance technologies currently available (1, 2). Amongst the wide variety of symptoms included in the term “urinary toxicity,” urinary incontinence (UI) plays an important role in the deterioration of patient quality of life.

The reported incidence of severe late incontinence after radical radiotherapy for PCa at 3–5 years ranges between 1 and 5%, but increases up to more than 20% in the post-operative setting (3–5). In general, since prostatectomy may negatively impact the urinary outcome *per se* (6), the actual detrimental impact on urinary function deriving from post-operative radiotherapy (PORT) is difficult to quantify.

The difficulty in sparing the bladder, owing to its proximity to the target, but especially the substantial lack of adequate knowledge concerning predictive factors of radiation-induced urinary incontinence represent the most significant limitations to further reducing both the rate and the severity of urinary complications. High-quality individually and prospectively collected data relative to a large number of patients followed for a sufficient long time are therefore eagerly awaited in order to develop reliable models in this field.

In addition, the optimal dose in the adjuvant (ART) and salvage (SRT) settings remains controversial. The radiation dose delivered after radical prostatectomy is typically 20–25% lower than that recommended in the case of radical radiotherapy (~60–64 *vs*. 76–80 Gy). More recently, the community of radiation oncologists has demonstrated a growing interest, supported both by elegant radiobiological models (7, 8) and retrospective analyses (7, 9, 10), in dose escalation in the setting of post-prostatectomy radiotherapy. The evidence of a relationship between radiation dose and clinical outcome has in fact been highlighted by several studies (8–12), supporting the hypothesis of a dose-response effect for PORT not significantly different from that observed for radical irradiation. The possible benefit deriving from escalating the radiation dose from 64 to 70 Gy in the salvage setting is currently under investigation by the randomized, multi-centric, Phase III trial SAKK 09/10 (13), whose preliminary results indicate that dose intensification of SRT had no impact on early urinary continence recovery or prevalence of *de novo* incontinence. Nevertheless, to date no robust data on a possible independent relationship between dose-escalation and effects of fractionation in the post-prostatectomy setting and an increased risk of mid-term risk of persistence/worsening of post-prostatectomy urinary incontinence are available, especially in the setting of patient-reported toxicity.

The main objectives of the current research were therefore:

1. the quantification of the dose-effect relationship of 2-year patient-reported urinary incontinence (PRUI) in the setting of post-prostatectomy irradiation;
2. the identification of clinically significant predictors of 2-year PRUI incidence after ART and SRT.

Urinary incontinence was assessed using patient-reported data prospectively collected within a prospective and registered multi-Institute observational study.

## Patients and Methods

### The IHU-WPRT TOX Study

The IHU-WPRT TOX (Intestinal Hematologic Urinary Toxicity from Whole-Pelvis Radiotherapy) is a registered multi-Institutional cohort study (ClinicalTrials.gov identifier #NCT02803086) aimed at developing predictive models of toxicity after WPRT for PCa (14–16). Before the activation of the IHU-WPRT TOX trial in February 2014, a Review Board approved pilot study had been performed at the Coordinating Institute (San Raffaele Scientific Institute, Milan, Italy) (14, 15). The IHU-WPRT TOX was approved by the Institutional Review Board of each of the participating Institutes and is still enrolling patients (16).

Prophylactic WPRT, always at the discretion of the referring radiation oncologist, is usually advised for patients with seminal vesicle invasion, Gleason score ≥ 7, pre-surgical PSA >10 ng/mL and/or histologically positive lymph-nodes at prostatectomy, or in the case of PSA ≥ 0.50 ng/mL in the salvage setting.

According to the protocol requirements (14–16), the validated Italian versions of the IBDQ (Inflammatory Bowel Disease Questionnaire) (17), ICIQ-SF (International Consultation on Incontinence Questionnaire-Short Form) (18) and IPSS (International Prostatic Symptom Score) (19) are to be prospectively collected for the patient-reported evaluation of WPRT-induced intestinal and urinary toxicity. All the questionnaires are to be filled in by the enrolled patients at baseline, at radiation treatment mid-point and end, at 3 and 6 months after radiotherapy conclusion, and thereafter every 6 months, up to 5 years. At identical time intervals a blood sample is to be collected for the evaluation of WPRT-induced hematologic toxicity (14, 15). In addition to clinical and dosimetric data (see below section Dose-Effect Quantification, Uni- and Multivariable Models and **Tables 3**, **4** for the list of the variables analyzed), patient personality and its possible impact on self-reported radiation-induced toxicities was considered by means of the abbreviated 24-item version of the revised Eysenck Personality Questionnaire (EPQ-R) (20) filled in by patients at baseline. Four personality traits are measured using four scales, each scored from 0 (no presence of the trait) to 6 (maximal presence of the trait): Extraversion (sociability, impulsiveness, but also some tendency to aggressiveness), Neuroticism (emotional instability, nervousness, and general anxiety), Psychoticism (tough-mindedness, but also a measurement of hostility) and Lie (a control scale introduced into personality measures in order to detect the “faking good” of scores on other scales; the Lie scale is reconstructed from items listing behaviors that are either socially desirable but infrequently practiced or frequently practiced but socially undesirable). When only one answer for each personality trait was missing (*n* = 12), imputation of EPQ-R scores was accomplished using the most frequent value reported by those patients who answered similarly.

### The ICIQ-SF Questionnaire

The ICIQ-SF consists of six items, three of which do not generate a score as they concern personal patient data (questions 1 and 2) or descriptive features (question 6). The quantitative items are represented by questions 3 (hereafter ICIQ3, score 0–5) and 4 (ICIQ4, score 0–6) pertaining to the frequency and amount of urine loss, respectively, and question 5 (ICIQ5, score 0–10) quantifying the subjective patient-perceived impairment of quality of life attributable to PRUI. The sum of these scores is used to quantify both the “objective” component (ICIQ3 + 4) and the “total” detriment (ICIQ3 + 4 + 5) of PRUI. Higher scores indicate worse symptoms.

### Patient Population

At the time of analysis, 2-year data were available for 213 patients. The population selected for the current work is composed of 71 patients from the pilot study (14, 15) and 142 from the observational protocol (16), with the requirements for the two studies being identical, according to the following criteria:

- patient underwent post-prostatectomy radiotherapy with either adjuvant or salvage intent.
- ICIQ-SF was completed both at baseline and at 24 months.
- at least two questionnaires were available between 6 and 24 months after radiotherapy end.

### ICIQ-Based Endpoints

The analysis focused on three clinically significant endpoints based on the quantitative and objective questions of the ICIQ-SF. In particular, endpoints were selected *a priori*, subsequent to a thorough discussion within the Institutes involved in the protocol, as those deemed to be “clinically significant” for patients, as follows:

- very frequent leakage (FREQUENCY), defined as an ICIQ3 score > 3 at least once between 6 and 24 months after PORT end;
- moderate to severe amount of urine loss (AMOUNT), defined as an ICIQ4 score >2 at least once between 6 and 24 months after PORT conclusion (the choice of this end-point, somewhat “weaker” as compared to that selected for FREQUENCY, was deemed necessary owing to the markedly lower number of events, see below);
- objective severe symptoms (OBJECTIVE), defined as the sum of ICIQ3 + 4 scores >5 at least once between 6 and 24 months following PORT end.

Incidentally, all of the three scores corresponded to the highest tertile in the considered population. For each endpoint only patients who did not exhibit an already impaired situation at baseline were considered, and therefore patients with ICQ3 > 3 or ICQ4 > 2 or ICQ3 + 4 > 5 at baseline were excluded when defining the corresponding endpoint.

A longitudinal analysis of the symptom trend across the 24 months post-radiotherapy was performed in order to characterize and compare the final sample group of patients and the part of population that was excluded from the statistical analyses.

### Dose-Effect Quantification, Uni-, and Multivariable Models

Firstly, a univariable logistic regression analysis was performed in order to select the best dose metrics associated to an increased risk of the ICIQ-based endpoints in terms of EQD2 doses calculated according to the different α/β values most commonly reported by the available literature for the bladder (i.e., 0.8, 3, and 5 Gy). The prescribed EQD2 to PBPTV was considered here as a surrogate for the high dose received by the normal bladder adjacent to the PBPTV. The dose metric corresponding to the model with the maximum log-likelihood was selected to be used for further analysis.

Univariable analyses were also performed in order to identify, for each endpoint, the most significant clinical variables, including age, body mass index (BMI, kg/m^2^), comorbidities such as diabetes and hypertension, smoking (yes *vs*. no/stopped at least 5 years before radiotherapy start), type of surgery, preoperative PSA, pathologic stage and Gleason score at prostatectomy, type and length of androgen deprivation therapy delivered concomitantly and after radiotherapy, months elapsed from prostatectomy to irradiation (time to radiotherapy), as well as ICIQ-SF scores at baseline.

Variables with a *p* < 0.2 at univariable analysis and without cross-correlations (Pearson or Spearman coefficient ∈[−0.25, 0.25]) were entered into a backward stepwise multi-variable logistic regression. Lastly, each model was reprocessed using only the variables retained by the backward multi-variable analysis with a *p*-value threshold ≤ 0.05.

Goodness of fit was assessed by means of the Hosmer and Lemeshow test and the calibration plot (slope and *R*^2^). Brier scores were used to measure accuracy. Internal validation was performed by 1,000 bootstrap resamplings, and optimism determined. Analyses were performed with MedCalc^®^ version 12.1.4.0 (MedCalc Software, Mariakerke Brussels, Belgium) and the R software version 3.2.4 (^©^The R Foundation for Statistical Computing, Vienna, Austria).

## Results

The patients were treated from 2011–2017 in 11 Italian Institutes with either conventionally-fractionated radiotherapy (*n* = 123, prescribed dose to PBPTV: 66.6–79.8 Gy in 1.8–2.0 Gy/fr.) or moderately hypo-fractionated regimens (*n* = 90, prescribed dose to PBPTV: 65.8–76.8 Gy in 2.1–2.7 Gy/fr.) with either adjuvant (*n* = 106) or salvage (*n* = 107) intent. Patient and treatment characteristics of the two subpopulations are detailed in Table 1.

**Table 1:** Summary of patient characteristics.

**Variables**	**Overall (*n =* 213)**	**Adjuvant (*n =* 106)**	**Salvage (*n =* 107)**
**PATIENT DATA**			
Age (yr)	67 (62–71)	67.5 (63–72)	67 (62–71)
BMI (kg/m2)	25.9 (15.9–27.7)	26.4 (24.4–28.1)	25.5 (24.1–27.3)
Hypertension (yes)	99 (47%)	49 (47%)	50 (47%)
Smoke (yes)	36 (17%)	21 (21%)	15 (14%)
Diabetes (yes)	15 (7%)	8 (8%)	7 (7%)
**SURGERY DATA**			
Type of Surgery			
Open	131 (63%)	74 (70%)	57 (55%)
Robotic	56 (27%)	23 (22%)	33 (32%)
Laparoscopic	22 (10%)	8 (8%)	14 (13%)
PSA (ng/ml)			
Pre-RP	8.01 (5.63–12.93)	8.68 (6.00–15.45)	7.60 (5.40–10.08)
Post-RP	0.04 (0.01–0.12)	0.07 (0.02–0.27)	0.03 (0.01–0.05)
No of removed lymph nodes	12 (6–20)	15 (8–22)	10 (3–18)
**RADIOTHERAPY DATA**			
PSA pre-RT (ng/ml)	0.24 (0.05–0.48)	0.06 (0.02–0.32)	0.33 (0.21–0.54)
Time to RT (mo)	8.3 (4–26.7)	4.0 (3.2–5.5)	26.7 (16.1–56.1)
Fractionation			
CONV (1.8–2.0 Gy/fr)	123 (58%)	55 (52%)	68 (64%)
HYPO (2.1–2.7 Gy/fr)	90 (42%)	51 (48%)	39 (36%)
Dose to PBPTV (Gy)			
Prescribed dose	71 (69–74)	70 (67–72)	73 (70–74)
EQD2(α/β = 0.8 Gy)	74 (70–76)	74 (70–74)	74 (70–76)
Irradiation technique			
SF-IMRT	20 (9%)	6 (6%)	14 (13%)
VMAT	106 (50%)	52 (49%)	54 (50%)
Tomotherapy	87 (41%)	48 (45%)	39 (37%)
Gleason score			
ISUP Groups 1–3	58 (27%)	17 (16%)	41 (39%)
ISUP Groups 4–5	154 (73%)	89 (84%)	65 (61%)
Stage T			
pT2	67 (32%)	11 (11%)	56 (52%)
pT3a	72 (34%)	36 (34%)	36 (34%)
pT3b & pT4	73 (34%)	58 (55%)	15 (14%)
**ANDROGEN DEPRIVATION THERAPY DATA**			
No ADT	116 (57%)	45 (45%)	71 (68%)
Bicalutamide monotherapy	25 (12%)	14 (14%)	11 (11%)
LH-RH	51 (25%)	35 (35%)	16 (16%)
CAB	12 (6%)	7 (7%)	5 (5%)
**PATIENT-REPORTED DATA**			
EPQ-R			
Extraversion	4 (3–5)	4 (3–5)	4 (3–5)
Neuroticism	1 (0–3)	1 (1–3)	1 (0–2)
Psychoticism	1 (0–1)	1 (0–1)	1 (0–2)
Lie	5 (4–6)	6 (5–6)	5 (4–6)
ICIQ-SF at baseline pre-RT			
ICIQ3			
Median	2	3	1
Quartiles (25–75%)	(0–4)	(1–4)	(0–3)
Tertiles (33–66%)	(1–3)	(2–4)	(0–2)
ICIQ4			
Median	2	2	2
Quartiles (25–75%)	(0–2)	(2–2)	(0–2)
Tertiles (33–66%)	(2–2)	(2–2)	(0–2)
ICIQ34			
Median	4	5	3
Quartiles (25–75%)	(0–6)	(3–6)	(0–5)
Tertiles (33–66%)	(3–5)	(4–6)	(0–4)

*Data are presented as counts (percentages in brackets) for categorical variables and as median values (inter-quartile ranges in bracket) for continuous variables. CONV, conventionally fractionated; HYPO, hypofractionated; BMI, body mass index; RP, radical prostatectomy; RT, radiotherapy; PBPTV, prostatic bed planning target volume; EQD2, 2-Gy equivalent dose; ADT, androgen deprivation therapy; CAB, combined androgen blockade; EPQ-R, eysenck personality questionnaire revised; ICIQ-SF, International Consultation On Incontinence Modular Questionnaire Short Form*.

The prescribed doses to PBPTV (*D*) were converted into 2-Gy equivalent doses (EQD2) according to the linear-quadratic model (21):

$$\begin{array}{l} EQD2\left(\alpha /\beta \right)=D\frac{\left(\alpha /\beta \;+\;d\right)}{\left(\alpha /\beta \;+\;2\right)} \end{array}$$

where *d* is the daily dose and α/β was set at 0.8, 3 and 5 Gy (hence EQD2(0.8), EQD2(3), EQD2(5), respectively), as reported in the literature (21, 22).

An impaired situation at baseline, according to the definition of the ICIQ-based endpoints (see ICIQ-Based Endpoints) was present in 31, 12, and 31% of patients for FREQUENCY, AMOUNT, and OBJECTIVE, respectively. As a consequence, the size of the final sample groups was: 148 patients for FREQUENCY, 188 for AMOUNT, and 148 for OBJECTIVE. It is noteworthy to underline that 31% of the entire population (67/213) was found to be “completely dry” (ICIQ3 + 4 = 0) before radiotherapy start.

**Figure 1:**
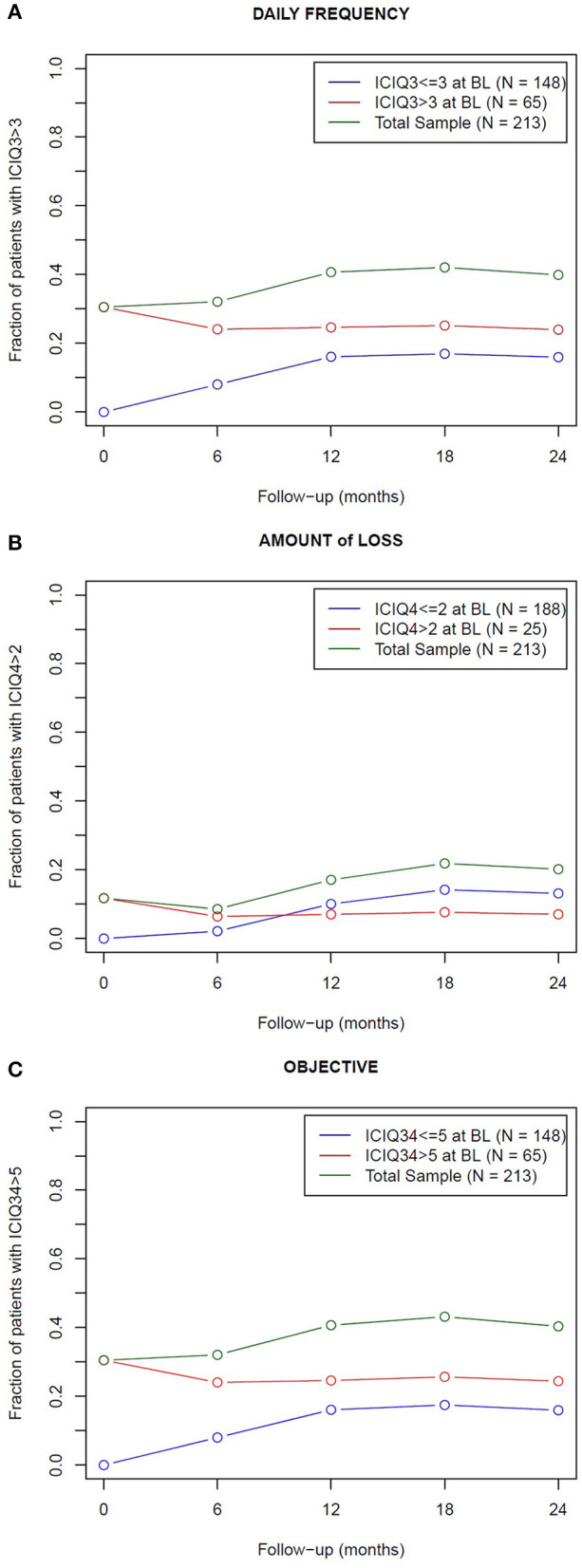
Fraction of patients who experienced endpoint symptoms (green curve) across the 24 months following post-prostatectomy radiotherapy. The total sample was dichotomized in patients who experienced (red curve) and did not experience (blue curve) the endpoint symptoms at baseline (BL): a not negligible fraction of patients with good baseline scores became incontinent (15–20%, according to the end-point definitions): inversely, the fraction of incontinent patients after radiotherapy slightly reduced in the group of patients incontinent at baseline. The endpoints are related to the frequency **(A)** and amount **(B)** of urine leakage and to the objective component **(C)** of urinary incontinence, as reported in the ICIQ-SF questionnaire.

**Figure 2:**
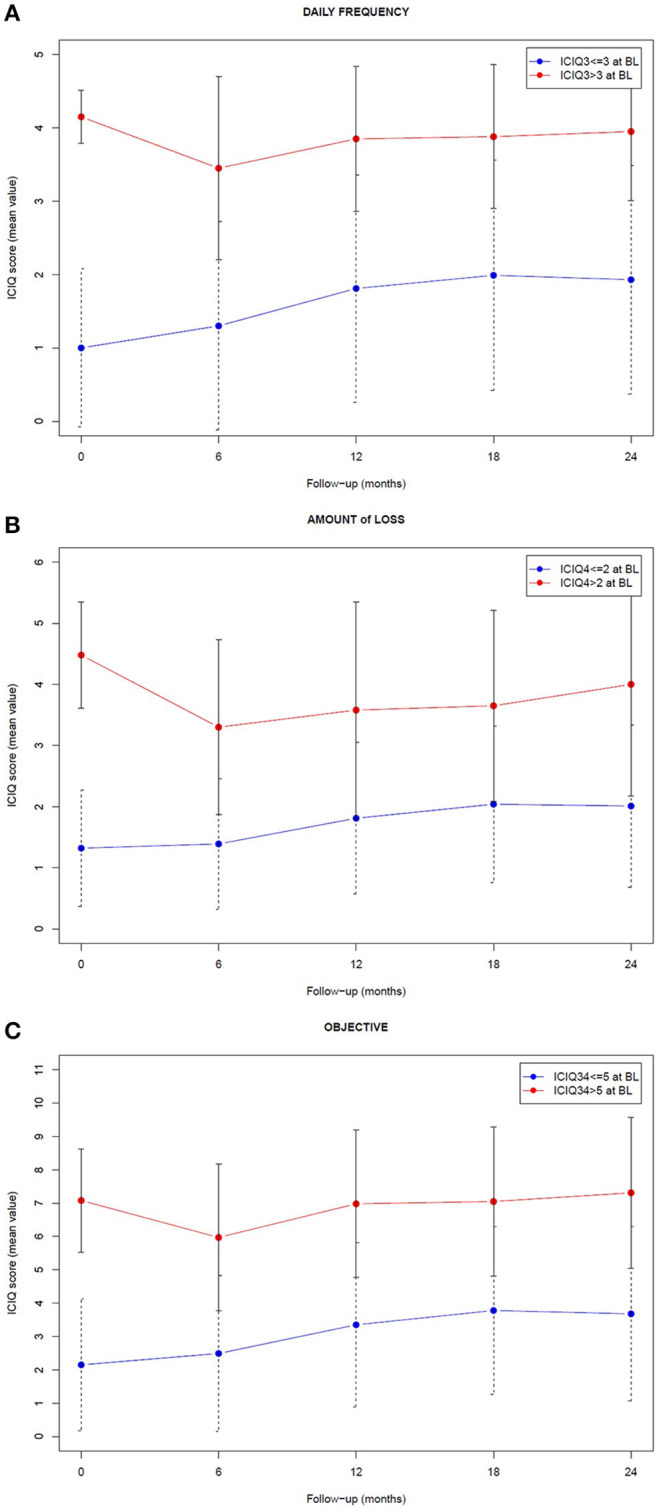
Longitudinal trend of the mean ICIQ score associated with the sample group of patients who experienced (red) and did not experience (blue) the endpoint symptoms at baseline (BL). The endpoints are related to frequency **(A)**, amount **(B)**, and objective component **(C)** of urinary incontinence, as reported in ICIQ-SF questionnaire. Error bars represent the standard deviation associated with the score distribution at each time.

The evolution of the fraction of patients experiencing (or not) the endpoint symptoms at baseline is shown in Figure 1, while the longitudinal trend of their mean ICIQ score is plotted in Figure 2. The Mann-Whitney-Wilcoxon test clearly indicated that the median values of the ICIQ score distributions in Figure 2 were always significantly different between the two sample groups (*p* < 0.001).

When looking to the differences from baseline scores, patients with good baseline scores (< ICIQ-based endpoints) showed a significant worsening 12 months after radiotherapy end and thereafter; patients with higher (= worse) baseline scores improved with the best recovery achieved 6 months after radiotherapy conclusion, then usually returned to the baseline incontinence level at 24 months (Table S1 of the Supplementary Material reports the p-values of the corresponding Mann-Whitney-Wilcoxon tests). In addition, the distribution of the changes between baseline and the 2-year scores confirmed an unbalanced distribution toward positive delta, i.e., worse symptoms in the group with good baseline score, as shown in Figure S1 of the Supplementary Material.

The results of univariable analyses are summarized in Tables 2, 3: an association with the dose was found for both the FREQUENCY and OBJECTIVE endpoints, while AMOUNT showed no dose-dependent trend (Table 2). The log likelihood of EQD2 (0.8) was higher with respect to those of EQD2 (3) and EQD2 (5); thus, EQD2 (0.8) was chosen as the dosimetric variable to be entered in the multi-variable analysis. For both FREQUENCY and OBJECTIVE endpoints, the *p*-values relative to EQD2 (0.8), age, smoking, and Gleason score were always *p* < 0.2, and the corresponding baseline ICIQ score emerged as the most significant variable (*p* < 0.001). The univariable analysis pertaining to the “amount of urine loss” endpoint (ICIQ4) confirmed baseline ICIQ as the most significant variable indicating, in addition, some role for age, robot surgery, and Extraversion (Table 3).

**Table 2:** Results of the univariable logistic regression analysis: association between the 2 Gy equivalent dose at different $\frac{\alpha }{\beta }$ values (0.8, 3, and 5 Gy) and the endpoints related to frequency, amount, and objective component of urinary incontinence, as reported in ICIQ-SF.

**Variables**	*****EQD***2(α/β)**		
	**α/β = 0.8 Gy**	**α/β = 3 Gy**	**α/β = 5 Gy**
**FREQUENCY endpoint (ICIQ3>3)**			
*p*-value	0.08	0.1	0.18
OR	1.06	1.09	1.09
CI (95%)	1.00–1.12	0.98–1.21	0.96–1.23
Log Likelihood	−94.32	−94.59	−95.02
**AMOUNTT endpoint (ICIQ4>2)**			
*p*-value	0.98	0.62	0.44
OR	1.00	1.03	1.05
CI (95%)	0.94–1.06	0.92–1.14	0.93–1.19
Log Likelihood	−97.31	−97.19	−97.02
**OBJECTIVE endpoint (ICIQ3+4>5)**			
*p*-value	0.08	0.1	0.18
OR	1.06	1.09	1.09
CI (95%)	1.00–1.12	0.98–1.21	0.96–1.23
Log Likelihood	−94.32	−94.59	−95.02

*ICIQ-SF, International Consultation On Incontinence Modular Questionnaire Short Form*.

**Table 3:** Results of the univariable logistic regression analysis (*p*-value and Odds-Ratio).

	**Frequency (ICIQ3>3)**		**Amount (ICIQ4>2)**		**Objective (ICIQ3+4>5)**	
**Variables**	***p*-value**	**OR**	***p*-value**	**OR**	***p*-value**	**OR**
**PATIENT VARIABLES**						
Age (yr)	**0.186**	1.04	**0.036**	1.06	**0.186**	1.04
BMI (kg/m2)	0.806	1.01	0.694	1.02	0.806	1.01
Hypertension						
No		Ref.		Ref.		Ref.
Yes	0.550	1.23	0.712	1.14	0.550	1.23
Smoke						
No		Ref.		Ref.		Ref.
Yes	**0.139**	1.95	0.855	0.91	**0.139**	1.95
Diabetes						
No		Ref.		Ref.		Ref.
Yes	0.752	1.24	0.878	1.11	0.752	1.24
**SURGERY VARIABLES**						
Type of Surgery						
Open		Ref.		Ref.		Ref.
Robotic	0.963	1.02	**0.186**	0.56	0.963	1.02
Laparoscopic	0.859	0.89	0.510	0.64	0.859	0.89
PSA (ng/ml)						
pre-RP	0.463	1.01	1.000	1.00	0.463	1.01
post-RP	0.846	0.97	0.928	1.02	0.846	0.97
No of removed lymph nodes	0.317	0.98	0.371	0.98	0.317	0.98
**RADIOTHERAPY VARIABLES**						
PSA pre-RT (ng/ml)	0.879	1.01	0.698	1.02	0.879	1.01
Time to RT (mo)						
≤ 8 months		Ref.		Ref.		Ref.
> 8 months	0.319	0.71	0.243	0.66	0.319	0.71
EQD2(α/β = 0.8Gy)	**0.076**	1.06	0.975	1.00	**0.076**	1.06
Gleason score						
ISUP Groups 1–3		Ref.		Ref.		Ref.
ISUP Groups 4–5	**0.159**	0.59	0.568	0.80	**0.159**	0.59
Stage T						
pT2		Ref.		Ref.		Ref.
pT3a	0.673	1.20	0.572	0.79	0.673	1.20
pT3b & pT4	0.294	1.55	0.221	0.57	0.294	1.55
**ANDROGEN DEPRIVATION THERAPY VARIABLES**						
ADT						
No		Ref.		Ref.		Ref.
Yes	0.714	0.88	0.440	1.32	0.714	0.88
**PATIENT-REPORTED VARIABLES**						
EPQ-R						
Extraversion	0.944	1.01	**0.089**	0.83	0.944	1.01
Neuroticism	0.762	1.03	0.205	1.15	0.762	1.03
Psychoticism	0.344	0.84	0.436	0.86	0.344	0.84
Lie	0.433	0.89	**0.145**	1.30	0.433	0.89
ICIQ-SF at baseline	**<0.001**	1.81	**0.006**	1.91	**<0.001**	1.39

*The endpoints are related to frequency, amount and objective component of urinary incontinence, as reported in ICIQ-SF. Significant values accepted for inclusion in subsequent multivariable analyses (p <0.2) are in bold. BMI, body mass index; RP, radical prostatectomy; RT, radiotherapy; ADT, androgen deprivation therapy; CAB, combined androgen blockade; EPQ-R, eysenck personality questionnaire revised; ICIQ-SF, International Consultation On Incontinence Modular Questionnaire Short Form*.

**Figure 3:**
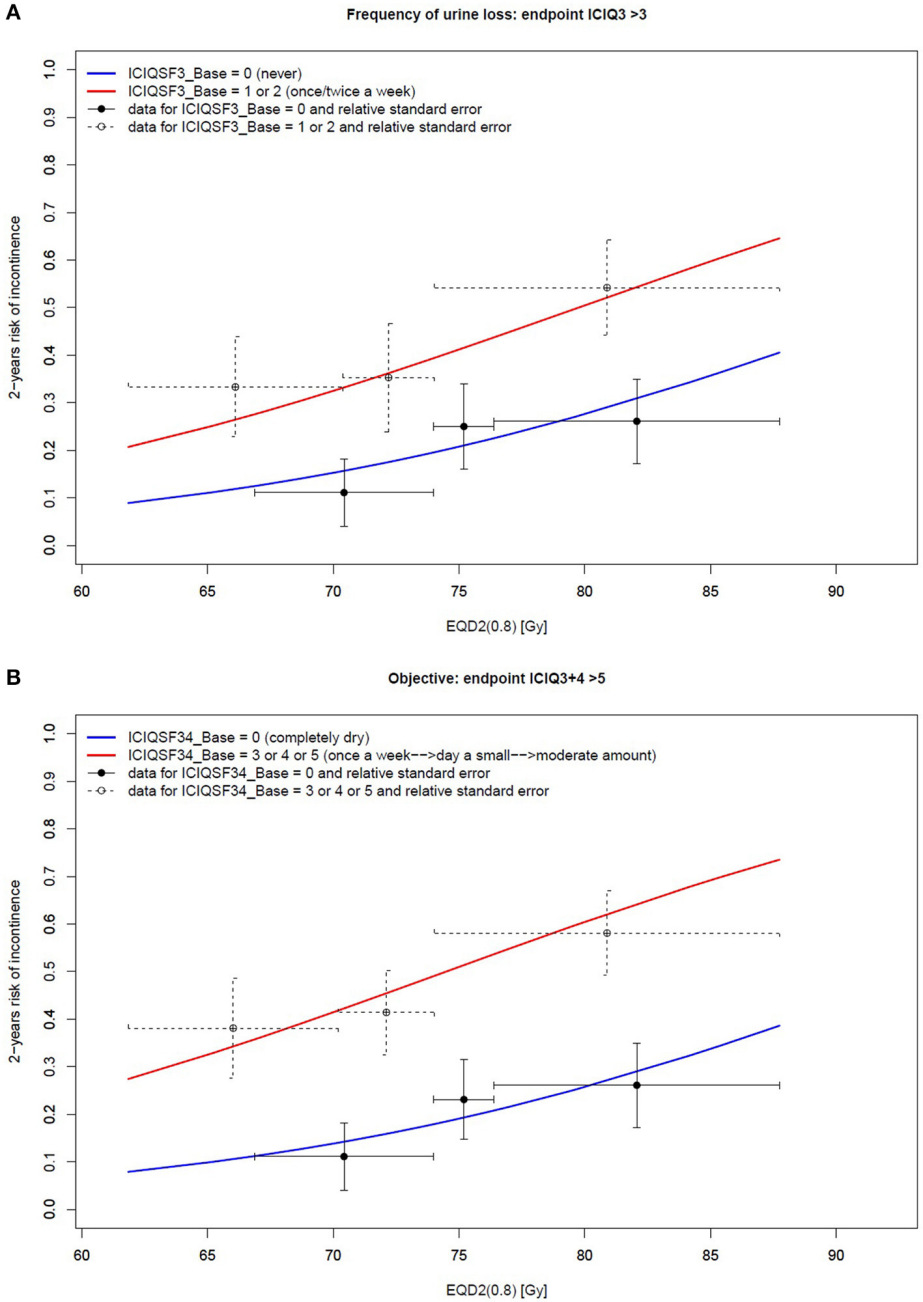
Two-variable model of the 2-year risk of urinary incontinence according to **(A)** the frequency of urine loss and **(B)** objective endpoints: the relationship between the dose and the ICIQ-SF score. Vertical bars represent the standard error, while horizontal bars represent the size of each tertile.

Table 4 outlines the resulting multi-variable models. Patients reporting none to mild symptoms at baseline experienced the pre-specified FREQUENCY, AMOUNT, and OBJECTIVE endpoints within 2 years from post-operative irradiation in 52/148 (35%), 40/188 (21%), and 52/148 (35%) of the cases, respectively. The corresponding baseline score was the most significant predictor for all endpoints (*p* < 0.006). Both FREQUENCY and OBJECTIVE were largely modulated by EQD2 (0.8), as illustrated in Figure 3.

**Table 4:** Results of the multi-variable logistic regression analysis.

**Predictors**	**Coeff ± dev.std**.	***p*-value**	**OR**	**CI (95%)**
**FREQUENCY—Frequency of urine loss**				
Endpoint: ICIQ3 >3, *N* = 52/148 (35%), excluded: ICIQ3 >3 at baseline				
Baseline score	0.655 ± 0.174	<0.001	1.93	1.38–2.74
EQD2(0.8) [Gy]	0.075 ± 0.033	0.024	1.08	1.01–1.15
Intercept	−6.964			
**H&L** = 0.78	**Slope** = 1.06	***R***^**2**^ = 0.82	**Brier score** = 0.198	
			(optimism = 0.01)	
**AMOUNT—Amount of Urine Loss**				
Endpoint: ICIQ4 >2, *N* = 40/188 (21%), excluded: ICIQ4 >2 at baseline				
Baseline score	0.648 ± 0.237	0.006	1.91	1.24–3.19
Intercept	−2.269			
**H&L** = 1.00	**Slope** = 1.00	***R***^**2**^ = 1.00	**Brier score** = 0.160	
			(optimism = 0.005)	
**OBJECTIVE—Objective**				
Endpoint: ICIQ3+4 >5, *N* = 52/148 (35%), excluded: ICIQ3+4 >5 at baseline				
Baseline score	0.371 ± 0.099	<0.001	1.45	1.20–1.77
EQD2(0.8) [Gy]	0.077 ± 0.033	0.022	1.08	1.01–1.16
Intercept	−7.220			
**H&L** = 0.97	**Slope** = 1.11	***R***^**2**^ = 0.92	**Brier score** = 0.199	
			(optimism = 0.009)	

*The endpoints are related to frequency, amount and objective component of urinary incontinence, as reported in ICIQ-SF*.

*H&L, Hosmer and Lemeshow test; ICIQ-SF, International Consultation on Incontinence Modular Questionnaire Short Form*.

**Figure 4:**
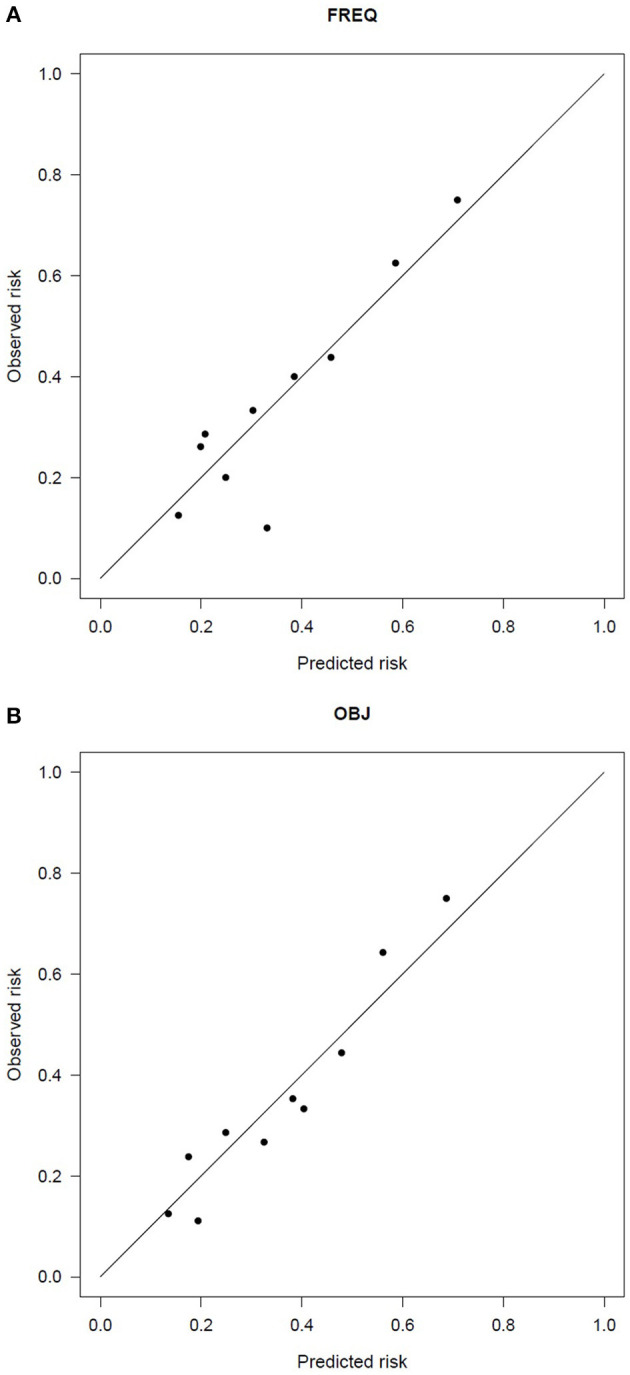
Calibration plot of the two-variable model of the 2-year risk of urinary incontinence according to **(A)** the frequency of urine loss and **(B)** the objective endpoints.

The goodness of fit was always satisfactory (Hosmer and Lemeshow test > 0.78), as was the calibration (see Figure 4), with slopes and *R*^2^ ranging between 1.0–1.1 and 0.82–1.00, respectively. Internal validation resulted in optimism of 0.005–0.01 on the Brier score, confirming the robustness of the results.

## Discussion

This is the first analysis focused on the identification of the main predictors of mid-term patient-reported UI and on the dose-effect quantification in a cohort of patients treated with post-prostatectomy irradiation. Owing to previous surgical injury, the urinary outcome of these patients could occasionally be somewhat worse than those undergoing radiotherapy with radical intent (23, 24); moreover, the growing evidence of a beneficial dose-effect relative to the risk of biochemical relapse (12) is gradually translating into the delivery of higher doses than in the past. The interplay between urinary recovery after surgery and the role of the timing of delivery of post-operative radiotherapy on baseline PRUI also contributes to further jeopardize the picture. Hence the necessity of a thorough analysis based upon both accurate endpoint definition and sufficiently large cohorts of patients treated at different dose levels in order to separately analyze the possible independent detrimental role of both previous surgery and dose-escalation in the post-prostatectomy setting.

The fine tuning of ICIQ-based endpoints allowed a focus on patients with none or only relatively mild symptoms after prostatectomy, corresponding to the most clinically significant subgroup, who exhibited a modulation and a worsening of patient-reported urinary incontinence across the 24 months following post-prostatectomy radiotherapy, as shown in Figures 1, 2. On the contrary, the mean ICIQ score of the remaining patients who began with severe symptoms did not increase (worsen) following post-surgical irradiation (Figure 2): interestingly, a significant improvement was observed at 6 months after radiotherapy (*p* < 0.002), probably as the result of a predominant recovery from surgical damage independent from radiotherapy. Furthermore, the difference between the ICIQ score distributions related to the groups with and without symptoms according to the different endpoint definition at baseline was always significant (*p* < 0.001), supporting the hypothesis that pre-radiotherapy baseline urinary incontinence is the strongest predictor of long-term post-prostatectomy patient-reported UI recovery, regardless of the subsequent delivery of adjuvant or salvage irradiation.

As baseline UI is expected to impact post-radiotherapy UI recovery, our results are also consistent with those of van Stam et al. (25), who observed that patients starting SRT seven months or more after RP were more likely to recover urinary function after irradiation (25). Both studies lend support to the growing trend to spare as many men as possible immediate adjuvant radiotherapy in order to permit full recovery of their post-prostatectomy UI, also taking into account the possibility of treating them safely with early salvage irradiation at the first sign of a PSA rise. The first results of the multicenter, randomized Phase III trials RADICALS, to be confirmed in a longer follow-up, indicate no difference in terms of 5-year biochemical relapse-free survival and freedom from salvage hormonal therapy between patients managed with immediate ART or early SRT. On the contrary, the incidence of 1-year patient-reported UI worsening with respect to baseline was slightly but significantly higher (5.3 vs. 2.7%, *p* = 0.008) in the cohort treated with immediate ART (26).

The high quality data of the IHU WPRT TOX database and the heterogeneous range of prescribed doses delivered with both conventional and moderately hypo-fractionated regimens allowed a thorough quantification of the dose effect. An independent correlation between the analyzed endpoints and EQD2 was found for FREQUENCY and OBJECTIVE, and the best fit was achieved using α/β = 0.8 Gy. These findings are strongly suggestive of an independent detrimental effect of both fractionation and higher doses on the risk of severe urinary incontinence following both adjuvant or salvage radiotherapy, consistent with the previously reported results of a large retrospective study (22).

Interestingly, the “amount” of urine leakage showed no relationship with the radiation doses, whereas it was found to be slightly correlated to one personality trait, Extraversion (*p* = 0.089) though at univariable analysis only. The apparent lack of dose-effect relationship for this endpoint could depend on the weak level of “objectivity” of the answers to the ICIQ4 item (the patient's perception of the “amount” of urine leakage is undoubtedly more “subjective” than that of “frequency”), as well as, at least in part, on the relatively “mild” end-point selected (ICIQ4 score >2 out of 6), a choice deemed necessary by the low frequency (21%) of more severe events. Consequently, the trend for frequency of urine loss (ICIQ3) also dominated the objective component of PRUI (ICIQ3 + 4) at both uni- and multivariable analyses.

The current analysis clearly highlighted that the probability of severe mid-term (2 years) PRUI was dramatically higher in patients with higher urinary incontinence baseline levels even when including only patients with none/mild symptoms at baseline, as in the current study. As shown in Figure 3, the 2-year risk of severe PRUI for patients with even only mild symptoms at baseline is much higher than that of the “completely dry” patients: when considering the range of doses typically delivered in the post-prostatectomy setting (65–75 Gy), this risk is in the range of 25–40% and 10–20%, respectively.

As recently reported, the 3-year risk for the same/slightly milder endpoints was around 5–10% for EQD2 (0.8) >80–85 Gy in the radical setting (3). This rate is dramatically lower than that found in this cohort of patients treated with post-prostatectomy radiotherapy, typically delivered at doses ≤ 75 Gy, even in the “completely dry” subset (as shown by the blue slope in Figure 3). These results suggest that, from the point of view of urinary incontinence, even in the case of an optimal baseline recovery at adjuvant or salvage radiotherapy start (i.e., “completely dry” patients) the clinical scenario is largely influenced by the “memory” of surgical injury, which is likely to negatively affect the repair capacity of the radiation-induced effects to the bladder and urethra.

## Conclusions

The most predictive factor of the 2-year risk of severe patient-reported urinary incontinence for patients treated with post-prostatectomy radiotherapy showing none to mild baseline symptoms was found to be the baseline level of urinary incontinence, showing that even mild incontinence symptoms are associated to an increased risk of 2-year severe incontinence. In addition, the frequency of urine loss was characterized by a marked dose-effect relationship that predominantly influenced the trend of the “objective” component (frequency + amount) of urinary incontinence; on this issue, the patient's perception of the “frequency” of urine loss seemed to be more reliable as an objective index than that of the “amount.” The identification of α/β = 0.8 Gy as the best fitting value confirmed previously reported findings from retrospective studies (22) and clearly highlighted the marked impact of fractionation on the risk of late severe urinary incontinence after post-prostatectomy radiotherapy.

## Data Availability Statement

The datasets generated for this study will not be made publicly available for privacy reasons.

## Ethics Statement

The studies involving human participants were reviewed and approved by San Raffaele Scientific Institute. The patients/participants provided their written informed consent to participate in this study.

## Author Contributions

CF and CC contributed to conception and design of the study. AB performed the statistical analysis and the interpretation of data under the supervision of CF and CC and wrote the first draft of the manuscript. All authors contributed to acquisition of data and to manuscript revision and approved the submitted version.

## Conflict of Interest

The authors declare that the research was conducted in the absence of any commercial or financial relationships that could be construed as a potential conflict of interest.
