# Supplementary material for: Predictors of 2-Year Incidence of Patient-Reported Urinary Incontinence After Post-prostatectomy Radiotherapy: Evidence of Dose and Fractionation Effects
Source: Front Oncol. 2020 Jul 23;10:1207. doi: 10.3389/fonc.2020.01207 (PMC7396712; doi:10.3389/fonc.2020.01207)
Supplement: Supplementary file 1 [file Data_Sheet_1.PDF]

Supplementary Material

Supplementary Table 1. Difference in terms of p-value between the ICIQ score distribution at baseline (BL) and those at each follow-up timing.

	**Wilcoxon Signed-Rank Test (p-values)**			
	**BL vs. 6 months**	**BL vs. 12 months**	**BL vs. 18 months**	**BL vs 24 months**
**Daily Frequency**				
ICIQ3 at BL ≤ 3	0.058	<0.001	<0.001	<0.001
ICIQ3 at BL > 3	0.000	0.018	0.033	0.147
**Amount of Losss**				
ICIQ4 at BL ≤ 2	0.623	<0.001	<0.001	<0.001
ICIQ4 at BL > 2	0.002	0.006	0.014	0.057
**Objective**				
ICIQ34 at BL ≤ 5	0.22	<0.001	<0.001	<0.001
ICIQ34 at BL > 5	0.000	0.347	0.958	0.644

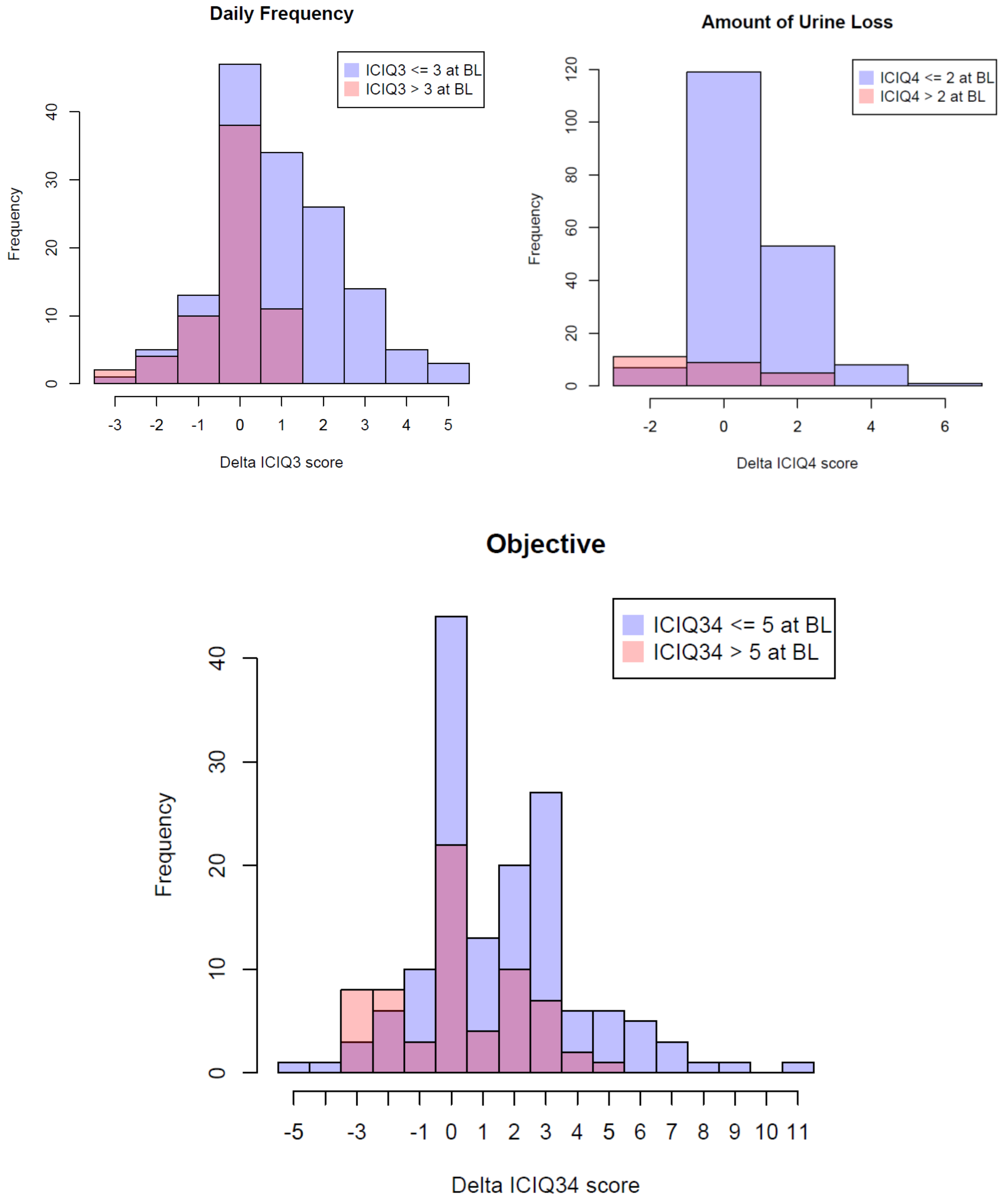

Supplementary Figure 1. Frequency distributions of delta ICIQ scores at 2 years for the groups of patients with/without endpoint symptoms at pre-radiotherapy baseline: a positive delta score means a worsening of the symptoms after 24 months from the post-prostatectomy irradiation.
